# Keel petal incision: a simple and efficient method for genetic crossing in *Medicago truncatula*

**DOI:** 10.1186/1746-4811-10-11

**Published:** 2014-05-16

**Authors:** Vijaykumar Veerappan, Khem Kadel, Naudin Alexis, Ashley Scott, Igor Kryvoruchko, Senjuti Sinharoy, Mark Taylor, Michael Udvardi, Rebecca Dickstein

**Affiliations:** 1 Department of Biological Sciences, University of North Texas, 1155 Union Circle #305220, Denton, Texas 76203, USA; 2 Plant Biology Division, The Samuel Roberts Noble Foundation, Ardmore, OK 73401, USA

**Keywords:** Legume, Genetic crossing, Barrel medic, *Medicago truncatula*, Artificial hybridization, Keel petal

## Abstract

**Background:**

Genetic crossing is an essential tool in both forward and reverse genetic approaches to understand the biological functions of genes. For *Medicago truncatula* (barrel medic) various crossing techniques have been used which differ in the methods used to dissect the female parent’s unopened flower bud to remove immature anthers for prevention of self-pollination. Previously described methods including front, side or back incision methods may damage the flower bud, impeding successful fertilization and/or seed development because they may allow pollen to dislodge and floral organs to desiccate after crossing, all of which diminish the success rates of crossing.

**Results:**

We report the keel petal incision method for genetic crossing in *M. truncatula* ecotype R108 and demonstrate successful crosses with two other *M. truncatula* ecotypes, A17 and A20. In the method presented here, an incision is made along the central line of the keel petal from the bottom 1/3rd of the female parent’s flower bud to its distal end. This allows easy removal of anthers from the flower bud and access for cross-pollination. After pollination, the stigma and the deposited pollen from the male donor are covered by the keel petal, wing petals and standard petal, forming a natural pouch. The pouch prevents dislodging of deposited pollen from the stigma and protects the internal floral organs from drying out, without using cling-film or water-containing chambers to maintain a humid environment. The keel petal incision method showed an approximate 80% success rate in the *M. truncatula* R108 ecotype and also in other ecotypes including Jemalong A17 and A20.

**Conclusions:**

Our keel petal incision protocol shows marked improvement over existing methods with respect to the ease of crossing and the percentage of successful crosses. Developed for the *M. truncatula* R108 ecotype, the protocol has been demonstrated with A17 and A20 ecotypes and is expected to work with other ecotypes. Investigators of varying experience have achieved genetic crosses in *M. truncatula* using this method.

## Background

*Medicago truncatula* (barrel medic) is an important model legume species extensively used to study symbiotic interactions with soil rhizobia and arbuscular mycorrhizal fungi leading to symbiotic nitrogen fixation and mycorrhization respectively [1-3]. It has also found use as a model for studies on secondary metabolism [4-6], plant pathogens [7,8], leaf development [9-11] and other processes. *M. truncatula*, in the Galegoid clade of the Papilionoideae legume subfamily, is closely related to economically important crops also in the Galegoid clade including alfalfa (*M. sativa*), pea (*Pisum sativum*) and lentil (*Lens culinaris*) as well as crop legumes in the Phaseoloid clade including soybean (*Glycine max*) and common bean (*Phaseolus vulgaris*) [12]. In the past two decades, extensive genomic and genetic resources have been developed for *M. truncatula*, making it an elite legume model amenable for functional genomics as well as for genetic studies to identify key regulators in important processes. These resources include a global gene expression atlas [13] and a nearly complete genome sequence [14]. *M. truncatula*’s genome may contain up to 50,000 protein coding genes, most of unknown biological function [14]. To facilitate genetic studies in *M. truncatula*, several populations of mutants have been developed [15], including those treated with ethyl methane sulfonate (EMS) [16], fast neutron bombardment (FNB) [17-19] and a near saturation insertion mutant population carrying the tobacco *Tnt1* retrotransposon [20-23].

An efficient technique for genetic crossing in *M. truncatula* is essential in our efforts to characterize mutants and to help identify their defective genes. Genetic crossing is commonly used to cross-fertilize mutants with their wild-type parents and to outcross mutants to different ecotypes to map the mutation of interest. Mutant populations generated using EMS, FNB or *Tnt1* transposition harbor multiple mutations in the same plant which may require backcrossing to the wild-type parent to remove extraneous mutations not linked to the mutant phenotypes under study and to investigate the mode of inheritance of phenotypes/traits. Back- and out-crossing provide valuable tools to identify causal mutations underlying mutant phenotypes by co-segregation/linkage analysis. The *M. truncatula* genome contains many gene families with redundant functions due to gene duplications [12]. To study the functions of redundant genes, double mutants can be created by genetic crossing [24]. Genetic crossing is also essential for linkage mapping of natural variations and for genome-wide association studies [25,26].

**Figure 1:**
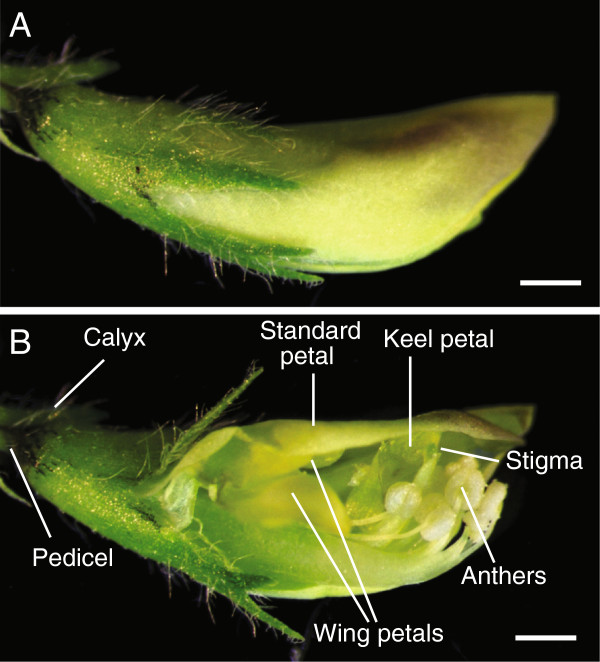
***M. truncatula *flower structure. A**, Unopened flower bud. **B**, Cut flower bud showing internal reproductive organs. Bars = 0.5 mm.

*M. truncatula* flowers are hermaphroditic, with both male and female organs in the same flowers. A *M. truncatula* flower is comprised of a calyx, a large standard petal, two small wing petals on both sides of the flower and a fused keel petal which cover both male and female floral organs (Figure 1A, B). The stigma is surrounded by eight fused anthers (Figure 1B). *M. truncatula* is a self-pollinated species and fertilization occurs when the flower is still closed (cleistogamy). When the flower reaches maturity, its anthers grow close to the stigma surface and rupture to release the pollen grains (anthesis) on the stigma surface which allows the flower to self-fertilize. When performing a genetic cross, it is important to artificially open the closed flower bud to visualize and access the anthers and stigma before anthesis. In a cross, for the flower bud serving as a female, all the anthers are removed before they release pollen. To artificially fertilize the female flower, pollen grains from mature anthers of a male donor are deposited onto the stigma surface of the female flower to allow cross-pollination to occur.

**Figure 2:**
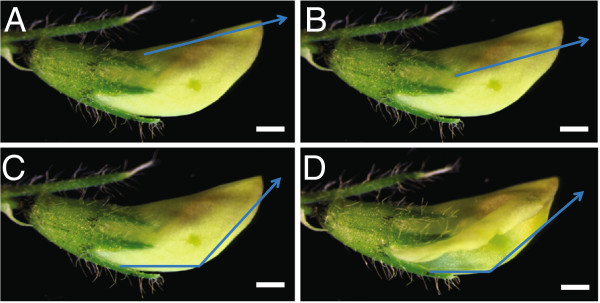
**Position of incisions used to artificially open the female flower bud to perform genetic crossing. A**, Front cut method. **B**, Side cut method. **C**, Back cut method. **D**, Keel petal incision method. Arrows indicate the sites of the cut/incision made on the unopened flower buds to perform anther removal and artificial pollination. Bars = 0.5 mm.

Several detailed genetic crossing techniques have been described for *M. truncatula*, which differ by how the immature flower bud is opened to allow removal of anthers and to allow access to the stigma for cross-pollination (Figure 2) [27-29]. These methods also differ in how the flower is treated post cross-pollination to assure a successful cross. We call these: (1) the front cut method [27-29]; (2) the side cut method, also called the pouch method [28,29]; and (3) back cut method (a variation of the side cut method; Mark Taylor, unpublished data). In the front cut method, the flower buds are cut vertically along the central line of the curved side of the standard petal towards its tip from the proximal one third of the flower bud (Figure 2A). In the side cut (Figure 2B) and back cut (Figure 2C) methods, similar cuts are made on the side and back of the flower buds respectively. The side cut method is time consuming and in all three procedures, the incision may damage the flower bud, rendering the female flower unusable. After cross-pollination using either the front or back cut methods, it is difficult to close the flower bud completely, which may lead to mechanical loss of deposited pollen and/or desiccation of internal floral organs.

Another crossing method employs male-sterile mutants as females. The *mtapetala* (*tap*) mutant, found in the *M. truncatula* Jemalong A17 ecotype [16], has been extensively used for this purpose [16,30-33]. However the *tap* mutation is not found in other ecotypes.

Here we report an improved genetic crossing technique called keel petal incision (Figure 2D; Additional file 1: Video S1; youtu.be/wDtRHWg1LBM), initially developed for the *M. truncatula* R108 ecotype. The keel petal incision method allows easy access to an immature flower for anther removal and artificial pollination. It also preserves floral morphological features permitting the standard and wing petals to close to their natural positions after cross pollination. This allows fertilization and embryo development to occur under protection of the flower petals and obviates the need for maintaining the pollinated flower in cling-film or humidified containers. Keel petal incision is a simple and efficient crossing technique with high success rates (>80%) in the R108 ecotype and in other ecotypes, A17 and A20.

## Results and discussion

### Plant growth and selection of optimal female and male flowers

**Figure 3:**
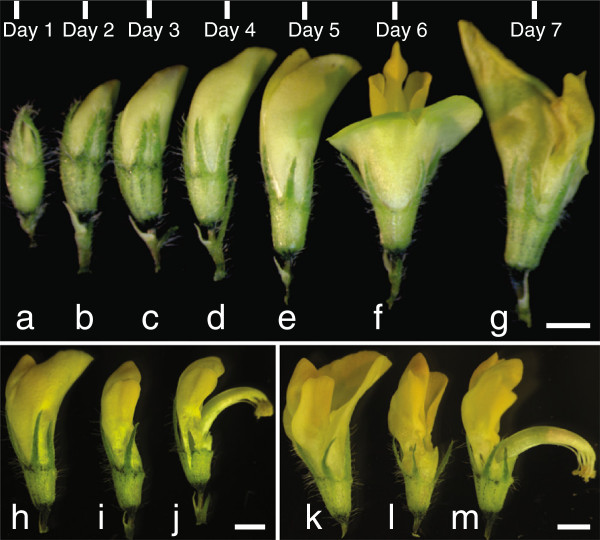
***M. truncatula *flower development and manipulation of pollen donor flowers. A-G**, Flowers from different developmental stages were collected, starting from when flower buds are first visible (Day 0). Flowers at day 3 are optimal to use as the female parent, while those at day 5 are best as the male parent. **H**, Day 5 flower. **I**, Day 5 flower with standard petal removed. **J**, Flower as in **I**, with stigma column exposed, showing anthers. **K**, Day 6 flower, rotated approximately 140° from flower in **F. L**, Day 6 flower with standard petal removed. **M**, flower as in **L**, rotated approximately 110°. Bar = 0.5 mm in top panel; bars = 1 mm in lower panels.

For the female in the crosses, we grew *M. truncatula* plants until they started flowering and began to develop one or two pods. Plants that have one or two developing pods will contain flowers in various stages of development as illustrated (Figure 3). For the female parent in the cross, very young flower buds (Figure 3A, B) are not optimal. While they have less chance of self-pollination than older flowers because they have immature anthers, they also typically have less mature stigmas as well. Older flowers (Figure 3D) have more mature stigmas, but also an increased likelihood of self-pollination caused by anther rupture during anther removal. Intermediate flower buds have more mature stigmas and not-yet ripe anthers (Figure 3C) and offer the best choice for a successful cross with fewer chances of self-pollination. For the male parent in the cross, flowers just about to open (Figure 3E, H) are the best choice. The stigma column and anthers can be triggered to open by applying slight pressure to the flower. Alternatively, fresh open (Figure 3F, K) flowers can serve as male parent/pollen donor. Rupture of anthers is verified under the dissecting microscope to make sure pollen grains are visible on the anther surface.

### Anther removal and artificial pollination

**Figure 4:**
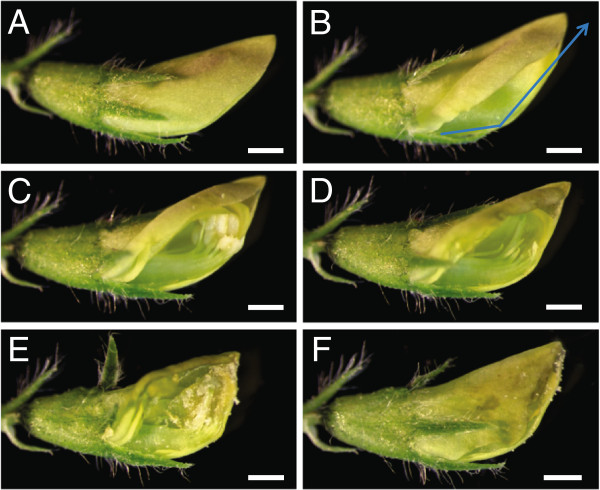
**Steps in genetic crossing using the keel petal incision method. A**, Unopened optimal female flower bud. **B**, Flower bud showing the keel petal under the standard petal indicating the site of incision on the central line of the keel petal. **C**, Flower bud showing the internal floral organs including anthers and stigma underneath the keel petal after making an incision. **D**, Flower bud showing stigma and style after the removal of anthers. **E**, Stigma covered with mature pollen after artificial cross-pollination. **F**, Closed flower bud after deposition of pollen on stigma. Bars = 0.5 mm.

After selecting an unopened flower bud as a female parent (Figure 3C), we removed the leaves, flowers, pods and/or shoots within three to four inches of the bud, leaving at most one trifoliate leaf adjacent to the chosen flower bud for ease of manipulation. Because *M. truncatula* flowers and their internal floral organs are small, it was not feasible to perform crosses using the naked eye. Hence, we performed crossing under a dissecting microscope; a magnifying glass or a magnifying binocular headset would also work for this purpose. The flower serving as female was mounted horizontally on its side on a dissecting microscope stage oriented so that the tip of the standard petal faced towards the base of the microscope and the opening of the standard petal faced the dominant hand of the person performing the cross (Figure 4A; Additional file 1: Video S1). The flower bud was secured to the microscope stage using cellophane tape on the pedicel. Subsequently, the flower bud was held in place using a set of forceps on the calyx, while another set of forceps was used to gently lift the standard petal to access the underlying keel petal. Using the sharp tip of a scalpel, the keel petal was gently cut at the bottom third of the flower bud and incision was made along the central ridge of the keel petal all the way to the distal end of the flower (Figure 4B). During this process, care was taken to assure that the tip of the scalpel blade cut only the keel petal and did not extend far inside the flower bud so as not to damage the stigma column underneath the keel petal and not to rupture the unopened anthers.

Subsequently, the half of the cut keel petal, facing the investigator, along with the wing and standard petals were pushed upwards with the forceps, allowing visualization and access to the anthers and stigma inside the flower bud (Figure 4C). At this point in preparing the flower for pollination, we checked whether the anthers were dehisced. If the anthers were already ruptured, we discarded that flower bud and chose another as the female crossing parent. If the anthers were not yet dehisced, we removed all eight of them by cutting the anther filaments away from the stigma column using sharp forceps tips. Alternatively, anther removal may be accomplished by suction using vacuum applied through a micropipette tip [16]. During anther removal, it is important not to rupture them and to avoid having the anthers contact the stigma. After anther removal, we ascertained that all the anthers were removed (Figure 4D) and visualized the tip of the stigma to verify that there was no accidental deposition of pollen grains on the stigma surface or inside the flower bud during anther removal. In the instances where we saw that this had occurred, we discarded that flower and started over with a fresh one.To expose the anthers from a male pollen donor in the cross, we triggered a flower that was about to open (Figure 3E, H) with gentle mechanical pressure until the style and anthers popped open. Alternatively, we have used forceps to remove the petals around the anthers in the pollen donor flower (Figure 3I). We found that using forceps to remove the standard petal first and then the wing and keel petals does not damage the anthers. Once the flower is triggered (Figure 3J) or the petals were removed, we observed the anthers using the dissecting microscope to ascertain that the anther was mature and dehisced with silvery white pollen grains released from ruptured anther sacs visible. This can also be seen using other magnification devices. Already-open flowers also served as pollen donors (Figure 3F, K). In these instances, the standard petal was removed with forceps (Figure 3L) exposing the style and anthers (Figure 3M; flower turned approximately 110° from Figure 3L).The mature anthers with attached pollen were then gently placed on the tip of the stigma of the female flower multiple times to deposit the pollen grains. At the end of this process, the entire tip of the stigma was surrounded by pollen (Figure 4E). There is variation in pollen quality and quantity from individual flowers; pollen quality may be negatively affected by insect infestation as well. In the case of insufficient pollen from one flower, we used more than one flower from the male pollen donor plant.After pollination, the flower was closed by pushing the top of the standard petal back into its original position to cover the wing petal, keel petal and stigma, forming a pouch around the stigma that looks similar to the unopened flower bud before crossing (Figure 4F). This prevented pollen from dislodging from the stigma during subsequent movement of the plant and protected the stigma and pollen from desiccation. The floral petal pouch obviates the need for protecting the pollinated flower with an artificial covering. These coverings promote fungal and bacterial growth on the flowers that may cause the flower to decay and drop. The pouch also does away with the need to place the cross-pollinated flower bud inside humid containers, which may also cause flower damage. After the cross-pollination was completed, the cellophane tape holding the pedicel was carefully removed and the pollinated flower was labeled.

### Seed pod development

**Figure 5:**
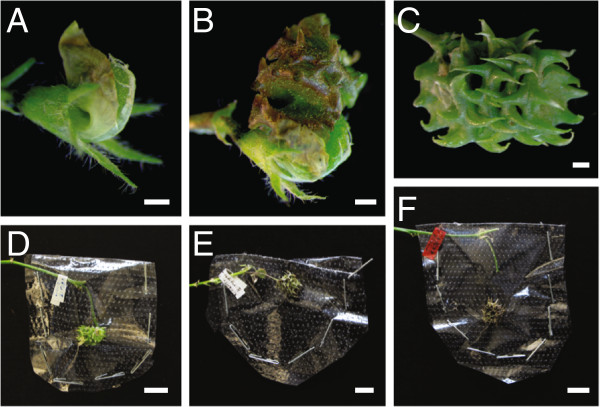
**Development of pods from successful crosses. A**, Curling of artificially cross-pollinated flower bud 3 days post hybridization. **B**, Developing pod 6 days post hybridization. **C**, Developing pod 20 days post-hybridization. **D**, Developing pod, 35 days post-hybridization wrapped with microperforated polythene sheet. **E**, Ripening yellow pod, 42 days post-hybridization. **F**, Completely matured darkened pod fallen off the pedicel, 50 days post-hybridization. Bars = 0.5 mm **(A-C)**. Bars = 5 mm **(D-F)**.

To distinguish between artificially pollinated and self-pollinated pods, we monitored the artificially pollinated flower buds daily and trimmed away new shoots growing adjacent to the pollinated flowers. Successful crosses were visualized by slight curling of pollinated flowers from the 3rd day onwards (Figure 5A). Most of the time, the cross-pollinated flowers continued to curl (Figure 5B) and develop into mature pods (Figure 5C). Once the pods from successful crosses developed into medium size (Figure 5C), we wrapped them using micro-perforated polythene sheets stapled in place (Figure 5D) to prevent the pods from falling off after maturity.To increase the size of the pods from cross-pollination and number of seeds per cross, we removed all pods on the plant resulting from self-pollination. Removing pods promotes more flower formation and prolongs the duration of flowering. After all the crosses were completed, shoots that do not carry pods from cross-pollination were trimmed. We observed developing pods turning yellow (Figure 5E) and darkening and dropping from the pedicel at maturity (Figure 5F).

We air-dried the pods from successful crosses at room temperature for 2–3 weeks. After this, we collected seeds from the pods and proceeded to confirm the crosses in the F_1_ generation. We found that larger pods typically contain 5–6 seeds/pod whereas the smaller pods only carried 1–3 seeds/pod. In general, crosses performed on younger plants resulted in larger pods whereas older plants produced smaller pods.

### Cross-pollination confirmation

As a first test of the success of cross-pollination by the keel petal incision method, we counted the number of seed pods that formed after cross pollination. We estimated an overall success rate of 82%, with slightly higher rates of pod formation when a wild-type parent was the female in the cross (Table 1).

**Table 1:** Estimation of crossing success via seed pod formation

**Crossing parents, wild-type as female**	**Attempted crosses**	**Seed pods formed**	**Success rate,%**
R108 X A17	9	6	67
R108 X A20	14	13	93
R108 X NF10796	17	12	71
R108 X NF11014	22	19	86
R108 X NF11044	27	22	81
R108 X NF11166	7	7	100
R108 X NF11217	11	8	73
R108 X NF1320	38	29	76
R108 X NF1320-29-3	17	17	100
R108 X NF1320-BC1-F3	21	16	76
R108 X NF4619	5	4	80
R108 X NF8324	31	23	74
A17 X R108	43	41	95
A17 X NF11044	91	82	90
A17 X NF11217	30	26	87
A17 X NF8324	18	12	67
A20 X R108	70	63	90
A20 X NF11044	58	49	84
A20 X NF11217	11	10	91
A20 X NF1320	15	9	60
A20 X NF8324	29	21	72
**Sub-total**	584	489	84
**Crossing parents, mutant as female**	**Attempted crosses**	**Seed pods formed**	**Success rate, %**
NF10796 X R108	14	9	64
NF11014 X R108	16	11	69
NF11044 X R108	28	22	79
NF11166 X R108	4	4	100
NF11217 X R108	16	11	69
NF1320 X R108	30	23	77
NF8324 X A20	7	5	71
NF8324 X R108	34	27	79
**Sub-total**	149	112	75
**Total crosses**	733	601	82

For each cross, the first parent listed in the cross served as the female. All crosses were performed by two graduate students, one with one year and the other with two years of research experience. Success rates were calculated based on whether a pod formed in the cross-pollinated flower buds ten days post-pollination.

Confirmation of successful crossing relied on phenotyping and genotyping the progeny. Our labs employ forward genetics to identify new legume genes that control the development of symbiotic nitrogen-fixing root nodules using the *M. truncatula Tnt1* insertion mutant collection in the R108 ecotype background. *Tnt1* mutants contain multiple *Tnt1* insertions and there are no naturally occurring *Tnt1* inserts in any *M. truncatula* ecotype [21,34]. To study the mode of inheritance of mutant phenotypes and to test the co-segregation of candidate *Tnt1* insertions with nodule phenotypes, we backcrossed two particular mutants with ineffective, white or brown non-fixing nodules (Nod + Fix-), NF11217 and NF10796, to their wild-type R108 using the keel petal incision method.

For NF11217 backcrosses, the mutant plant was used as the female parent and the wild-type R108 served as the male pollen donor. For NF10796 backcrosses, wild-type served as the female and the mutant plant was used as a male parent. Typically, it is advisable to perform reciprocal crosses by switching male and female parents in separate crosses, because the mutant phenotypes might be governed by maternal or paternal inheritance.

**Figure 6:**
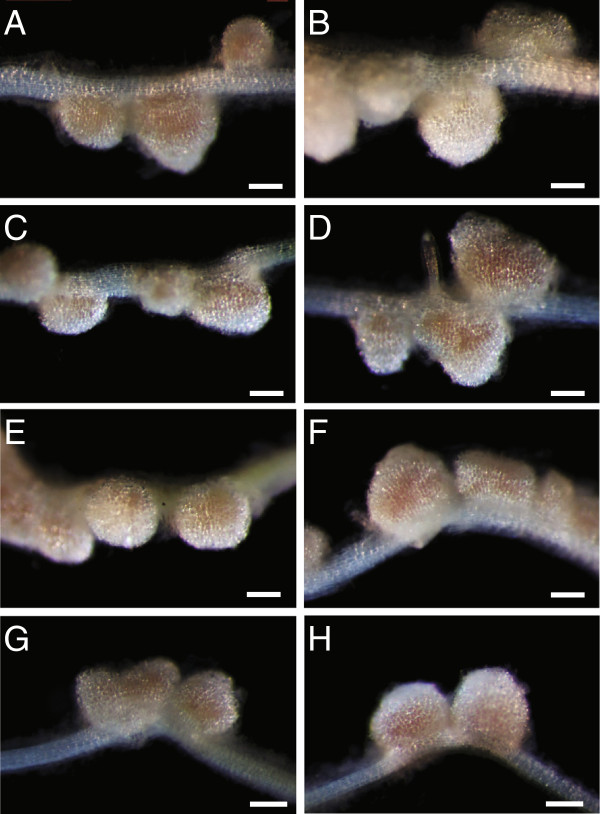
**Confirmation of successful crosses by nodule phenotypes of the progeny. ***M. truncatula* plants were grown on an aeroponic chamber, as described in Materials and methods. Five days after germination, plants were starved for nitrogen for five days and then inoculated with *Sinorhizobium meliloti*. Root nodules were examined 15 days post-inoculation. **A**, Wild-type R108. **B**, NF11217 mutant. **C-H**, NF11217 X wild-type R108-BC_1_F_1_. The R108 plant **(A)** has pink wild-type nodules while those of NF11217 are white and ineffective **(B)**. Each plant from the BC_1_F_1_ progeny **(C-H)** has pink nodules indicative of a successful cross. Bars = 250 μm.

When recessive *Tnt1* mutants were used as female, successful crosses were confirmed by wild-type phenotype progeny in the F_1_ population. Nodules of NF11217 mutant (female parent), wild-type (male parent) and NF11217 × wild-type-BC_1_F_1_ progeny were assessed for visual phenotypes fifteen days after inoculation of roots with *Sinorhizobium meliloti*. The nodules from wild-type parents are pink (Figure 6A) because of the presence of leghemoglobin associated with efficient symbiotic nitrogen fixation, whereas the NF11217 mutant plants show white nodules, defective in symbiotic nitrogen fixation (Figure 6B). All the BC_1_F_1_ plants tested showed pink nodules similar to the wild-type (Figure 6C-H), indicating that cross-pollination was successful and suggesting the mutation in NF11217 is recessive.

**Figure 7:**
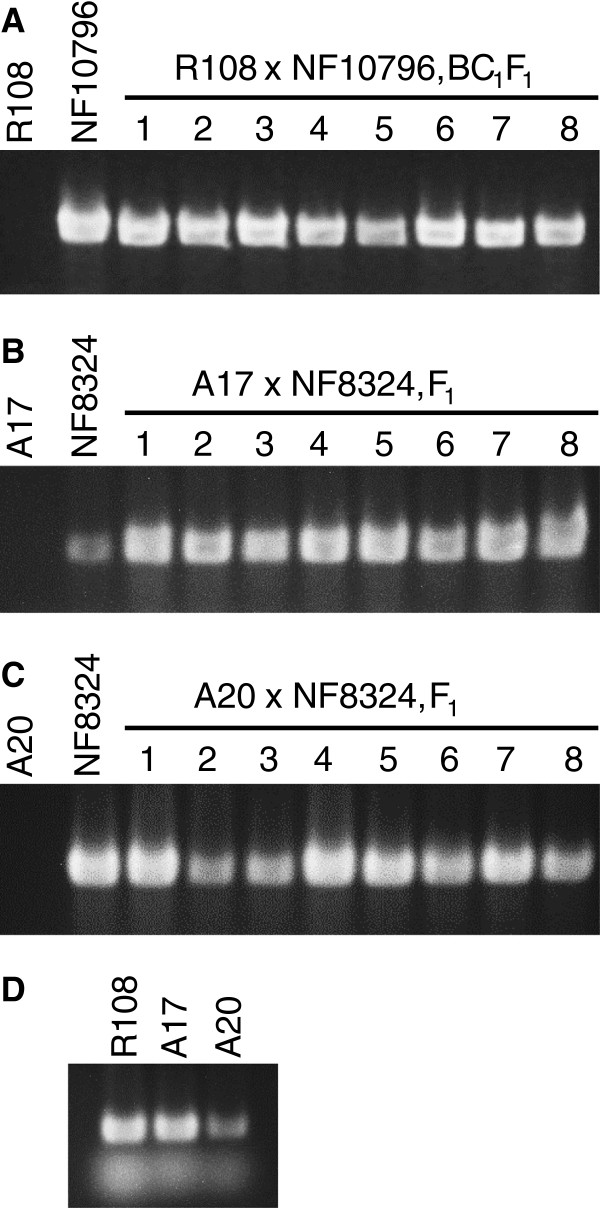
**Confirmation of successful crosses by genotyping. A-C**, Wild-type R108, NF10796 and NF8324, R108 x NF10796-BC_1_F_1_, A17 x NF8324-F_1_ and A20 x NF8324-F_1_ plants were PCR genotyped using *Tnt1* transposon specific primers. **D**, Primers for the *MtIRE-like* gene were used in the PCR amplifications as positive control for the wild-type genomic DNA. *Tnt1* DNA is not found in wild-type R108, A17 or A20 plants but can be detected in all F_1_ plants indicating successful crosses **(A-C)**. Stained gel images have been cropped to show the PCR products of 612 bp from *Tnt1* and 422 bp from *MtIRE-like* amplifications respectively.

When wild-type was used as female, success of the crosses was verified by genotyping the F_1_ plants for the presence of *Tnt1* transgene. For the case of NF10796 serving as the male in a cross with a wild-type R108 female, PCR (polymerase chain reaction) genotyping of BC_1_F_1_ progeny using *Tnt1* specific primers was performed. Our results showed the presence of *Tnt1* insertions in the R108 x NF10796-BC_1_F_1_ plants (Figure 7A) indicating crossing success. As expected, there was no amplification of *Tnt1* sequences in the wild-type R108 (Figure 7A).

As a further test of the utility of the keel petal incision method, we attempted out-crossing of *Tnt1* Nod+ Fix- mutant NF8324 in the R108 background, to both the A17 and A20 wild-type ecotypes. In these cases, we used the A17 or A20 wild-types as females and NF8324 as a male in the crosses. F_1_ plants arising from the crosses were genotyped by PCR for the presence of *Tnt1* sequences in the progeny, indicating a successful cross. As can be seen by the examples (Figure 7B, C), the keel petal incision method also worked for these other *M. truncatula* ecotypes. However, F_1_ progeny from inter-ecotype crosses involving R108 were observed to have developmental phenotypes (not shown); thus, special care must be taken with these progeny.

We examined ninety BC_1_F_1_ plants for nodulation phenotypes resulting from backcrosses involving Nod+/Fix- mutants female parents with wild-type R108 as male and found almost all of them had wild-type nodules, indicating successful cross-pollination and absence of self-pollination (Table 2). With the reciprocal situation using the wild-type as female and mutant as male, we tested forty plants from the progeny and obtained success rates of approximately 87% overall using the R108, A17 and A20 ecotypes, as verified by presence of *Tnt1* sequences in the BC_1_F_1_ and F_1_ progeny (Table 2).

**Table 2:** Confirmed successful crosses

**Crossing parents, mutant as female**	**Total number of plants tested**	**Number of confirmed plants by nodule phenotyping**	**Success rate,%**
NF11217 X R108	28	28	100
NF11044 X R108	31	31	100
NF8324 X R108	17	17	100
NF1320 X R108	14	11	78
**Crossing parents, wild-type as female**	**Total number of plants tested**	**Number of confirmed plants by PCR genotyping**	**Success rate,%**
R108 X NF10796	8	8	100
R108 X NF8324	13	11	84
A17 X NF8324	8	8	100
A20 X NF8324	11	8	72

For each cross, the first parent listed in the cross was used as the female. For cases where the mutant served as female in the cross, successful crossing was confirmed by the appearance of pink nodules in the BC_1_F_1_ progeny. When wild-type served as female, including inter-ecotype crosses, crosses were confirmed in the BC_1_F_1_, or F_1_ progeny by PCR genotyping using *Tnt1* specific primers, as in Figure 7.

## Conclusions

Crossing is essential for genetics. Here we described a new crossing technique for *M. truncatula* that involves incising the keel petal of the female flower to expose the flower’s stigma and anthers, remove its anthers and permit an artificial cross. After the cross is complete, the standard, wing and keel petals can be arranged similarly to their original positions in the unopened flower bud, protecting the floral organs from desiccation and removing the need for extraneous materials to maintain humidity for the fertility of the flower and deposited pollen. We demonstrated that this method can be used for the *M. truncatula* R108, A17 and A20 ecotypes and expect that it will find utility in other ecotypes. This method has been successfully used by investigators of varying experience, including junior graduate students and undergraduates in our labs.

## Materials and methods

### Plant growth

*M. truncatula* seeds were scarified with sulfuric acid, treated with bleach, imbibed and vernalized as described [16,28]. Shorter vernalization (1–4 days at 4°C) results in delayed flowering and a larger plant with higher numbers of flowers. Longer vernalization (4–14 days at 4°C) results in earlier flowering accompanied by smaller plant stature and reduced flower numbers. If plants are grown exclusively for crossing, longer vernalization is preferred to shorten the time to flowering. Details of the effect of vernalization on flowering time and growth stature can be found in [28]. Different vernalization times were used and did not appear to affect crossing success. After vernalization, seeds were germinated at 25°C in the dark; in our hands, seeds germinated within 12–24 hours. Subsequently, the plants were screened for nodulation phenotypes 15 days post-inoculation with *S. meliloti* using an aeroponic chamber [30,35], or by growing the plants on a Turface/vermiculite mixture [23]. After phenotyping, the seedlings were grown in medium size (four inch; ten cm) pots so that they are easy to handle while performing crosses. To avoid waterlogged roots, we grew *M. truncatula* in a peat-based potting medium with Turface (Profile, Buffalo Grove, Ill) mixed in (3:1), at 22°C under16:8 hr light:dark cycles. Plants were irrigated as needed and fertilized with a commercial general purpose fertilizer once a week.

### Crosses and PCR confirmation

The crosses were performed under a Nikon model C-PS stereo microscope (Nikon, Tokyo, Japan). Images and video clips of *M. truncatula* flowers and crossing techniques were obtained using Leica MZ10F (Leica, Buffalo Grove, IL) microscope. Two pairs of fine tip forceps (HL-14 #5, http://www.buyincoins.com) and a straight-edge scalpel (scalpel blade handle 9303 #3, and scalpel blade 9311 #11, both from http://www.microscopesamerica.com) were used for keel petal incision, the removal of anthers from the unopened female flower bud and artificial cross-pollination (Additional file 2: Figure S1). Mature pods from the successful cross-pollinations were wrapped using micro-perforated polythene sheets (MP1120160T, http://www.prismpak.com).

The following primers were used for PCR genotyping to confirm crosses: *Tnt1*-F, GCATTCAAACTAGAAGACAGTGCTACC and *Tnt1*-R, TGTAGCACCGAGATACGGTAATTAACAAGA [34] (*Tnt1*, Genbank:X13777). *MtIRE-like* specific primers [36] (*MtIRE-like*, Genbank:AY770392, Genbank:AC122727) were used as a control to confirm that extracted DNA from wild-type plants was of sufficient quality for PCR using *MtIRE-*F, CCAAATCGTTGAAAGCTCGTTCACAACTCC and *MtIRE-*R, CGTCTTGACCAGCAAACACGACACG.

## Abbreviations

BC: Back cross; DNA: Deoxyribonucleic acid; EMS: Ethyl methane sulfonate; FNB: Fast neutron bombardment; *M. truncatula*: *Medicago truncatula*; PCR: Polymerase chain reaction.

## Competing interests

The authors declare that they have no competing interests.

## Authors’ contributions

VV designed the experiments and made the supplemental video. VV, KK, NA, AS, IK, SS and MT carried out the experiments and edited the manuscript. MU edited the manuscript. VV and RD coordinated the experiments, and wrote and edited the manuscript. All authors read and approved the final manuscript.

## Supplementary Material

Additional file 1: Video S1 The keel petal incision crossing method for *M. truncatula*. The method is narrated step-by-step: youtu.be/wDtRHWg1LBM. Click here for file

Additional file 2: Figure S1 Forceps and scalpel used in crossing. Two pairs of fine tip forceps, e.g., HL-14 #5, http://www.buyincoins.com, and a straight-edge scalpel, e.g., scalpel blade handle 9303 #3, and scalpel blade 9311 #11, both from http://www.microscopesamerica.com, were used for keel petal incision, the removal of anthers from the unopened female flower bud and cross-pollination. Similar forceps and scalpels are available from other vendors. Bar = 5 cm. Click here for file

## References

[B1] Cook DR *Medicago truncatula* - a model in the making! Curr Opin Plant Biol 1999 2 301 304 10.1016/S1369-5266(99)80053-3 10459004

[B2] Kouchi H Imaizumi-Anraku H Hayashi M Hakoyama T Nakagawa T Umehara Y Suganuma N Kawaguchi M How many peas in a pod? Legume genes responsible for mutualistic symbioses underground Plant Cell Physiol 2010 51 1381 1397 10.1093/pcp/pcq107 20660226 PMC2938637

[B3] Barker DG Bianchi S London F Dattee Y Duc G Essad S Flament P Gallusci P Genier G Muel X Tourneur J Denarie J Huguet T *Medicago truncatula*, a model plant for studying the molecular genetics of the *Rhizobium*-legume symbiosis Plant Mol Biol Report 1990 8 40 49 10.1007/BF02668879

[B4] Dixon RA Liu C Jun JH Metabolic engineering of anthocyanins and condensed tannins in plants Curr Opin Biotechnol 2013 24 329 335 10.1016/j.copbio.2012.07.004 22901316

[B5] Verdier J Zhao J Torres-Jerez I Ge S Liu C He X Mysore KS Dixon RA Udvardi MK MtPAR MYB transcription factor acts as an on switch for proanthocyanidin biosynthesis in *Medicago truncatula* Proc Natl Acad Sci USA 2012 109 109 10.1073/pnas.1120916109 PMC3277187 22307644

[B6] Zhao J Dixon RA The ‘ins’ and ‘outs’ of flavonoid transport Trends Plant Sci 2010 15 72 80 20006535 10.1016/j.tplants.2009.11.006

[B7] Uppalapati SR Ishiga Y Doraiswamy V Bedair M Mittal S Chen J Nakashima J Tang Y Tadege M Ratet P Chen R Schultheiss H Mysore KS Loss of abaxial leaf epicuticular wax in *Medicago truncatula irg1/palm1* mutants results in reduced spore differentiation of anthracnose and nonhost rust pathogens Plant Cell 2012 24 353 370 10.1105/tpc.111.093104 22294617 PMC3289574

[B8] Uppalapati SR Marek SM Lee H-K Nakashima J Tang Y Sledge MK Dixon RA Mysore KS Global gene expression profiling during *Medicago truncatula–Phymatotrichopsis omnivora* interaction reveals a role for jasmonic acid, ethylene, and the flavonoid pathway in disease development Mol Plant Microbe Interactions 2009 22 7 17 10.1094/MPMI-22-1-0007 19061398

[B9] Tadege M Lin H Bedair M Berbel A Wen J Rojas CM Niu L Tang Y Sumner L Ratet P McHale NA Madueño F Mysore KS *STENOFOLIA* regulates blade outgrowth and leaf vascular patterning in *Medicago truncatula* and *Nicotiana sylvestris* Plant Cell 2011 23 2125 2142 10.1105/tpc.111.085340 21719692 PMC3160033

[B10] Lin H Niu L McHale NA Ohme-Takagi M Mysore KS Tadege M Evolutionarily conserved repressive activity of WOX proteins mediates leaf blade outgrowth and floral organ development in plants Proc Natl Acad Sci USA 2013 110 366 371 10.1073/pnas.1215376110 23248305 PMC3538250

[B11] Ge L Peng J Berbel A Madueño F Chen R Regulation of compound leaf development by *PHANTASTICA* in *Medicago truncatula* Plant Physiol 2014 164 216 228 10.1104/pp.113.229914 24218492 PMC3875802

[B12] Zhu H Choi H-K Cook DR Shoemaker RC Bridging model and crop legumes through comparative genomics Plant Physiol 2005 137 1189 1196 10.1104/pp.104.058891 15824281 PMC1088312

[B13] Benedito VA Torres-Jerez I Murray JD Andriankaja A Allen S Kakar K Wandrey M Verdier J Zuber H Ott T Moreau S Niebel A Frickey T Weiller G He J Dai X Zhao PX Tang Y Udvardi MK A gene expression atlas of the model legume *Medicago truncatula* Plant J 2008 55 504 513 10.1111/j.1365-313X.2008.03519.x 18410479

[B14] Young ND Debellé F Oldroyd GED Geurts R Cannon SB Udvardi MK Benedito VA Mayer KFX Gouzy J Schoof H Van de Peer Y Proost S Cook DR Meyers BC Spannagl M Cheung F De Mita S Krishnakumar V Gundlach H Zhou S Mudge J Bharti AK Murray JD Naoumkina MA Rosen B Silverstein KAT Tang H Rombauts S Zhao PX Zhou P The *Medicago* genome provides insight into the evolution of rhizobial symbioses Nature 2011 480 520 524 22089132 10.1038/nature10625 PMC3272368

[B15] Tadege M Wang TL Wen J Ratet P Mysore KS Mutagenesis and beyond! Tools for understanding legume biology Plant Physiol 2009 151 978 984 10.1104/pp.109.144097 19741047 PMC2773078

[B16] Penmetsa RV Cook DR Production and characterization of diverse developmental mutants of *Medicago truncatula* Plant Physiol 2000 123 1387 1398 10.1104/pp.123.4.1387 10938356 PMC59096

[B17] Wang HL Li G Chen R Silva JT Fast Neutron Bombardment (FNB) Mutagenesis for Forward and Reverse Genetic Studies in Plants Floriculture, Ornamental and Plant Biotechnology: Advances and Topical Issues (1st Edition). Volume 1 2006 Isleworth, UK: Global Science Books 629 639

[B18] Rogers C Wen J Chen R Oldroyd G Deletion-based reverse genetics in *Medicago truncatula* Plant Physiol 2009 151 1077 1086 10.1104/pp.109.142919 19759346 PMC2773085

[B19] Rogers C Oldroyd GED Kahl G, Meksem K Fast Neutron Mutagenesis for Functional Genomics The Handbook of Plant Functional Genomics 2008 Weinheim, Germany: Wiley-VCH 291 306

[B20] Tadege M Ratet P Mysore KS Insertional mutagenesis: a Swiss Army knife for functional genomics of *Medicago truncatula* Trends Plant Sci 2005 10 229 235 10.1016/j.tplants.2005.03.009 15882655

[B21] Tadege M Wen J He J Tu H Kwak Y Eschstruth A Cayrel A Endre G Zhao PX Chabaud M Ratet P Mysore KS Large-scale insertional mutagenesis using the *Tnt1* retrotransposon in the model legume *Medicago truncatula* Plant J 2008 54 335 347 10.1111/j.1365-313X.2008.03418.x 18208518

[B22] Cheng X Wen J Tadege M Ratet P Mysore KS Reverse genetics in *Medicago truncatula* using *Tnt1* insertion mutants Methods Mol Biol 2011 678 179 190 10.1007/978-1-60761-682-5_13 20931380

[B23] Pislariu CI Murray JD Wen J Cosson V Muni RRD Wang M Benedito VA Andriankaja A Cheng X Jerez IT Mondy S Zhang S Taylor ME Tadege M Ratet P Mysore KS Chen R Udvardi MK A *Medicago truncatula* tobacco retrotransposon insertion mutant collection with defects in nodule development and symbiotic nitrogen fixation Plant Physiol 2012 159 1686 1699 10.1104/pp.112.197061 22679222 PMC3425206

[B24] Floss DS Levy JG Lévesque-Tremblay V Pumplin N Harrison MJ DELLA proteins regulate arbuscule formation in arbuscular mycorrhizal symbiosis Proc Natl Acad Sci USA 2013 doi:10.1073/pnas.1308973110 10.1073/pnas.1308973110 PMC3870710 24297892

[B25] Yeoh CC Balcerowicz M Zhang L Jaudal M Brocard L Ratet P Putterill J Fine mapping links the *FTa1* flowering time regulator to the dominant *Spring1* locus in Medicago PLoS One 2013 8 e53467 doi:53410.51371/journal.pone.0053467 10.1371/journal.pone.0053467 23308229 PMC3538541

[B26] Jaudal M Yeoh CC Zhang L Stockum C Mysore KS Ratet P Putterill J Retroelement insertions at the Medicago *FTa1* locus in *spring* mutants eliminate vernalisation but not long-day requirements for early flowering Plant J 2013 76 580 591 10.1111/tpj.12315 23964816

[B27] Pathipanawat W Jones RAC Sivasithamparam K An improved method for artificial hybridization in annual *Medicago* species Aust J Agric Res 1994 45 1329 1335 10.1071/AR9941329

[B28] Chabaud M Lichtenzveig J Ellwood S Pfaff T Journet E-P Mathesius U, Journet E-P, Sumner LW Vernalization, Crossings and Testing for Pollen Viability The Medicago Truncatula Handbook 2006 http://www.noble.org/MedicagoHandbook/. ISBN 0-9754303-1-9

[B29] Taylor M Blaylock L Nakashima J McAbee D Ford J Harrison M Udvardi M Mathesius U, Journet E-P, Sumner LW *Medicago Truncatula* Hybridization – Supplemental Videos The Medicago Truncatula Handbook, Version 2011 2011 Ardmore, Oklahoma, USA: The Samuel Roberts Noble Foundation 1 5 vol. http://www.noble.org/MedicagoHandbook/. pp. 1–5

[B30] Veereshlingam H Haynes JG Sherrier DJ Penmetsa RV Cook DR Dickstein R *nip*, a symbiotic *Medicago truncatula* mutant that forms root nodules with aberrant infection threads and plant defense-like response Plant Physiol 2004 136 3692 3702 10.1104/pp.104.049064 15516506 PMC527167

[B31] Schnabel E Mukherjee A Smith L Kassaw T Long S Frugoli J The *lss* supernodulation mutant of *Medicago truncatula* reduces expression of the *SUNN* gene Plant Physiol 2010 154 1390 1402 10.1104/pp.110.164889 20861425 PMC2971615

[B32] Schnabel EL Kassaw TK Smith LS Marsh JF Oldroyd GED Long SR Frugoli JA The *ROOT DETERMINED NODULATION1* gene regulates nodule number in roots of *Medicago truncatula* and defines a highly conserved, uncharacterized plant gene family Plant Physiol 2011 157 328 340 10.1104/pp.111.178756 21742814 PMC3165882

[B33] Penmetsa RV Frugoli JA Smith LS Long SR Cook DR Dual genetic pathways controlling nodule number in *Medicago truncatula* Plant Physiol 2003 131 998 1008 10.1104/pp.015677 12644652 PMC166865

[B34] D’Erfurth I Cosson V Eschstruth A Lucas H Kondorosi A Ratet P Efficient transposition of the *Tnt1* tobacco retrotransposon in the model legume *Medicago truncatula* Plant J 2003 34 95 106 10.1046/j.1365-313X.2003.01701.x 12662312

[B35] Journet E-P de Carvalho-Niebel F Andriankaja A Huguet T Barker DG Mathesius U, Journet E-P, Sumner LW Rhizobial Inoculation and Nodulation of *Medicago Truncatula* The Medicago Truncatula Handbook 2006 http://www.noble.org/MedicagoHandbook/. ISBN 0-9754303-1-9

[B36] Pislariu CI Dickstein R An *IRE*-like AGC Kinase Gene, *MtIRE*, has unique expression in the invasion zone of developing root nodules in *Medicago truncatula* Plant Physiol 2007 144 682 694 10.1104/pp.106.092494 17237187 PMC1914176

