# Supplementary material for: Keel petal incision: a simple and efficient method for genetic crossing in Medicago truncatula
Source: Plant Methods. 2014 May 16;10:11. doi: 10.1186/1746-4811-10-11 (PMC4070640; doi:10.1186/1746-4811-10-11)
Supplement: Additional file 1: Video S1 — The keel petal incision crossing method for M. truncatula. The method is narrated step-by-step: youtu.be/wDtRHWg1LBM. [file 1746-4811-10-11-S1.pdf]

Supplemental video for: Keel petal incision: A simple and efficient method for genetic crossing in *Medicago truncatula*, Vijaykumar Veerappan, Khem Kadel, Naudin Alexis, Ashley Scott, Igor Kryvoruchko, Senjuti Sinharoy, Mark Taylor, Michael Udvardi, Rebecca Dickstein

There is a supplemental video for this manuscript that is available at the following link: http://youtu.be/wDtRHWg1LBM . The video is unlisted and only those with the link can see it.

(The file is currently over the 250 MB file size limit for upload.)
