# Supplementary material for: Keel petal incision: a simple and efficient method for genetic crossing in Medicago truncatula
Source: Plant Methods. 2014 May 16;10:11. doi: 10.1186/1746-4811-10-11 (PMC4070640; doi:10.1186/1746-4811-10-11)
Supplement: Additional file 2: Figure S1 — Forceps and scalpel used in crossing. Two pairs of fine tip forceps, e.g., HL-14 #5, http://www.buyincoins.com, and a straight-edge scalpel, e.g., scalpel blade handle 9303 #3, and scalpel blade 9311 #11, both from http://www.microscopesamerica.com, were used for keel petal incision, the removal of anthers from the unopened female flower bud and cross-pollination. Similar forceps and scalpels are available from other vendors. Bar = 5 cm. [file 1746-4811-10-11-S2.pdf]

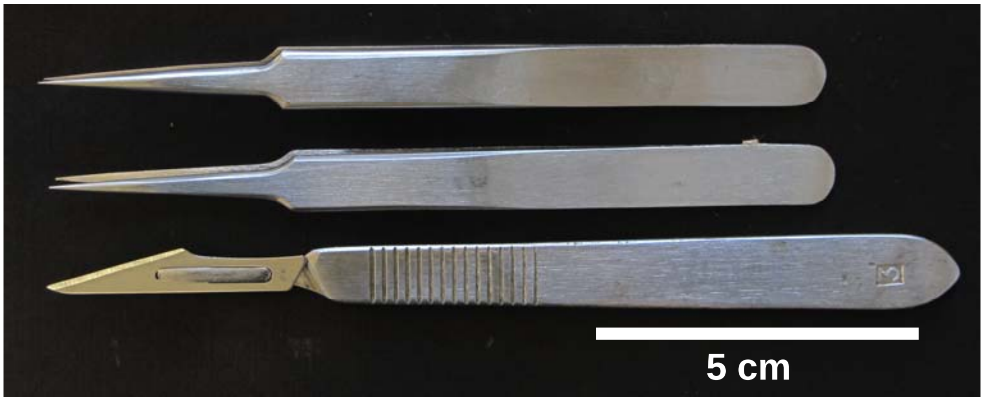

Figure S1. Forceps and scalpel used in crossing. Two pairs of fine tip forceps, e.g., HL-14 #5, www.buyincoins.com, and a straight-edge scalpel, e.g., scalpel blade handle 9303 #3, and scalpel blade 9311 #11, both from www.microscopesamerica.com, were used for keel petal incision, the removal of anthers from the unopened female flower bud and cross-pollination. Similar forceps and scalpels are available from other vendors. Bar=5cm.
